# The reachability of contagion in temporal contact networks: how disease latency can exploit the rhythm of human behavior

**DOI:** 10.1186/s12879-018-3117-6

**Published:** 2018-05-15

**Authors:** Ewan Colman, Kristen Spies, Shweta Bansal

**Affiliations:** Department of Biology, Georgetown University, Washington, 20057 DC USA

**Keywords:** Reachability, Contact network, Transmission, Synchronization, Circadian rhythm, Latent period, Generation time

## Abstract

**Background:**

The symptoms of many infectious diseases influence their host to withdraw from social activity limiting their potential to spread. Successful transmission therefore requires the onset of infectiousness to coincide with a time when the host is socially active. Since social activity and infectiousness are both temporal phenomena, we hypothesize that diseases are most pervasive when these two processes are synchronized.

**Methods:**

We consider disease dynamics that incorporate behavioral responses that effectively shorten the infectious period of the pathogen. Using data collected from face-to-face social interactions and synthetic contact networks constructed from empirical demographic data, we measure the reachability of this disease model and perform disease simulations over a range of latent period durations.

**Results:**

We find that maximum transmission risk results when the disease latent period (and thus the generation time) are synchronized with human circadian rhythms of 24 h, and minimum transmission risk when latent periods are out of phase with circadian rhythms by 12 h. The effect of this synchronization is present for a range of disease models with realistic disease parameters and host behavioral responses.

**Conclusions:**

The reproductive potential of pathogens is linked inextricably to the host social behavior required for transmission. We propose that future work should consider contact periodicity in models of disease dynamics, and suggest the possibility that disease control strategies may be designed to optimize against the effects of synchronization.

**Electronic supplementary material:**

The online version of this article (10.1186/s12879-018-3117-6) contains supplementary material, which is available to authorized users.

## Background

The prevention and mitigation of infectious disease outbreaks remains a major challenge that crosses several scientific disciplines. While advances in virology and immunology remain crucial for the development of vaccines and therapeutics, contributions from the social and behavioral sciences continue to improve our understanding of disease transmission at the population scale [1, 2]. Recent work in the field of network epidemiology has made important strides in our understanding of how the dynamics of individual and population social behavior drive epidemic outbreaks [3–8]. However, many questions still remain about the complex interplay that emerges from coupling the dynamics of human social behavior with the life-cycle of a pathogen [9].

After entering a human host, diseases typically experience a *latent period*. During this time, the pathogen multiplies within the host, but is yet unable to transmit to others. This is then followed by the *infectious period*, during which disease transmission is possible [10]. The latent period is closely related to the *incubation period* (the time between receiving the infection and the onset of symptoms), but in many cases infectiousness precedes symptoms, resulting in a period of time for which the host is capable of transmission but unaware that they have been exposed to the infection [11, 12].

An additional aspect of disease dynamics is the behavioral responses of the host and the community following the onset of illness. *Sickness behaviors* such as fever, lethargy, depression, and loss of appetite, cause the host to limit their movement and social interactions [13–16]. These responses are thought to be adaptive as they protect host resources, reduce pathogen reproduction, and promote inclusive fitness by protecting the social group from infection [17]. In humans, transmission can be reduced significantly through social isolation or through the avoidance behavior of other susceptible individuals [18, 19].

**Fig. 1:**
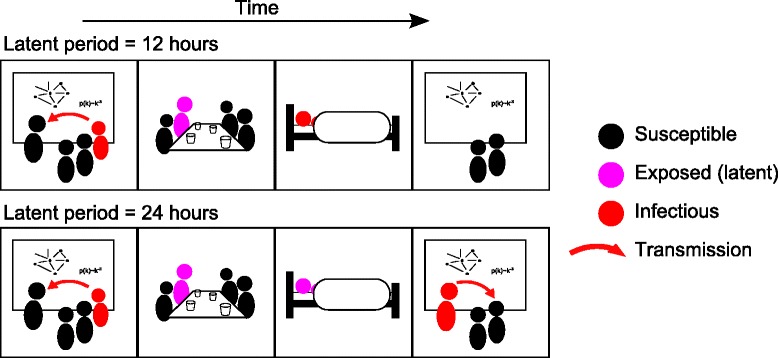
Conceptual illustration showing the effect of different latent periods. In the upper panel, the host becomes infectious 12 h after receiving the infection, at which point he has entered a more sedentary phase of his daily schedule. The symptoms of the infection influence him to avoid returning to his school or workplace and no further transmission occurs. In the lower panel, the infectious period begins at the same time of the day that he received the infection. While the symptoms of the disease may result in social withdrawal, there is a period of time for which he is both infectious and socially active, giving the disease an opportunity to spread

Here, we focus on the effect of social withdrawal behaviors on the spread of disease. While previous studies have considered reduced or rewired contacts [20, 21], we choose to examine the possibility that the infectious period, which is typically estimated from survey data or controlled experiments [12, 22, 23], is effectively cut short by the onset of sickness behaviors. Under these conditions, it may be possible for diseases to have a reproductive advantage when their latent period synchronizes with the timing of human social behavior (see Fig. 1).

To test this hypothesis, we use temporally resolved human contact network data from various social settings (a conference, a school, and a hospital) and ask how disease spread would fare in each of these networks. We also consider this question in semi-empirical networks which include both household and non-household contacts. We primarily focus on diseases that have a short window of opportunity for transmission to occur; this assumes a rapid reduction in social contact after the onset of infectiousness and therefore may not be appropriate for less virulent infectious diseases.

Biological rhythms are known to influence within-host infection dynamics, immune response, and transmission between hosts [24, 25]. There has been recent interest in the idea that pathogens can adapt to take advantage of periodic changes to their environment. For example, viruses whose life-cycle synchronizes with the timing of regular antibiotic treatment may be more resistant to the drug than those who do not [26]. Our work is a contribution to this growing area with a focus on the interaction between host social behavior cycles and pathogen dynamics.

## Methods

The goal of our study is to use empirical contact data to reveal the presence and magnitude of the synchronization between contact dynamics and disease progression. While the underlying concept may be relevant to a range of infectious diseases, we focus specifically on a moderately-transmissible respiratory disease such as influenza. After describing the empirical data used in this study we introduce a method to measure the contagion potential of a temporal contact network with respect to a disease with given latent and infectious period durations. We then describe a method to quantify the effect of synchronization for simulated disease spread on a temporal contact network, and lastly outline how we couple the disease simulation with three different host behavioral responses.

### Data

In the first two parts of our analysis we use human contact data from the Sociopatterns project (sociopatterns.org), and in the third part, we utilize synthetic networks based on demographic data from an urban area [27].

Data relating to the kind of close-proximity interactions that allow respiratory diseases to transmit was downloaded from the Sociopatterns website. The data has the format of a temporal network, meaning that for each interaction recorded, we are given the identities of the two individuals involved, and the times for which the interaction started and ended. We use human contact data recorded in three separate locations and Sociopatterns studies. Participants wore radiofrequency identification (RFID) sensors that detect face-to-face proximity of other participants within 1−1.5 meters in 20-s intervals. Each data-set lists the identities of the people in contact, as well as the 20-s interval of detection. To exclude contacts detected while participants momentarily walked past one another, only contacts that are detected in at least two consecutive intervals are considered interactions.

**Fig. 2:**
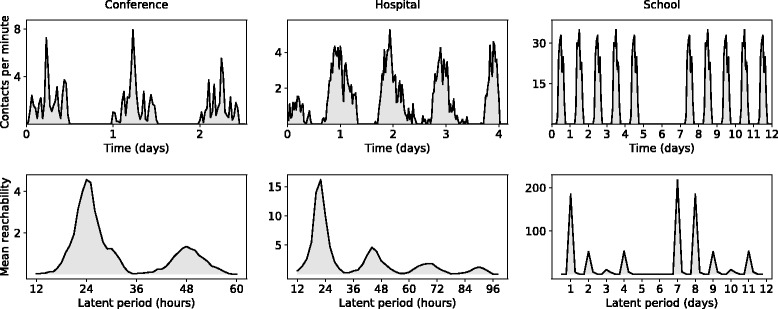
Reachability. The top panels show the number of face-to-face interactions between pairs of individuals (in the school data we only show 2 of the 6 weeks). The bottom panel shows the mean reachability of a disease over a range of latent periods. For the purpose of presentation we have subtracted the number of nodes reached directly from the seed (this is the same for all values of the latent period duration). The tendency for latent periods which are larger multiples of 24 h or 7 days to result in lower reachability is explained by the limited time span of the data-sets

Sensors were not worn outside the locations being studied so there are long spans of inactivity corresponding to the periods from early evening to early morning (See the top panels of Fig. 2). The three data-sets used were: (a) a conference in which 110 participants were recorded over 3 days [28], (b) a hospital ward in which 74 participants were recorded over 4 days [29], and (c) a primary school in which 242 participants were recorded over 2 days [30, 31]. In a similar fashion to the original study [30], we looped this data to produce a dataset spanning six weeks. We used the first day to represent Monday, Wednesday and Friday and the second day to represent Tuesday and Thursday. We then added 2 days of inactivity to replicate a typical school week and weekend.

Because the data provided by each of the above studies is limited to a single setting, we used another data approach to consider the impact of more complete networks, including home contacts in addition to the non-home contacts captured in the datasets discussed above. We used the procedure used in [27] (originally developed in [32]) to generate synthetic contact networks based on empirical distributions of ages, household sizes, school and classroom sizes, hospital occupancy, workplaces, and public spaces from the Greater Vancouver Regional District.

We generated a population with 1094 households, which yielded 2692 individuals. Each member of this population was assigned to activities according to their age, and edges between individuals were created based on their location and nature of their overlapping daily activities. Full details of how edges were assigned can be found in [27]. For the present study we converted this static network into a temporal network by adding times to the edges in the following way: we synthesize a typical weekday by assigning to all non-household contacts a start time at 8am and end time at 4pm and to household contacts a start time of 4pm and end time of 12am. A typical weekend day is created by assigning a start time of 8am and end time of 12am to all household contacts. We then piece these days together to create six weeks of temporal network data.

### Measuring disease impact through reachability

The *reachability* of an individual, *X*, is defined as the number of individuals that could potentially become infected given that *X* is the source of infection [33–36]. The original definition applied only to contagion processes where each individual can be in one of only two states: susceptible or infectious (i.e. an SI model). The concept has since been developed to incorporate the recovered state (i.e. an SIR model) [37]. Here, we have further extended the concept of reachability to include a latent state of infection (i.e. an SEIR model).

We compute the reachability of *X* with respect to a given set of disease parameters; namely, the latent period, *Δ*_*E*_, which is the length of time from when an individual is exposed to infection to the time they are able to transmit to others (more commonly called the “Exposed” state); and the effective infectious period, *Δ*_*I*_, which is defined as the length of time a host remains infectious after the latent period has elapsed. To capture the impact of sickness behaviors, we will assume very short durations for *Δ*_*I*_. The host may experience illness for longer (the true infectious period, which we denote *Δ*_*J*_) but, due to the lack of social interactions, there are no transmission events possible after *Δ*_*I*_.

We say that a member of the population, *X*, can “reach” another, *Z*, if a sequence of contacts exists that makes it feasible for a pathogen to spread from *X* to *Z*. For this to be the case, the time between any two consecutive contacts in the sequence must be greater than *Δ*_*E*_ and less than *Δ*_*E*_+*Δ*_*I*_ (see Figure S1 in the Additional file 1). We impose one further restriction that the sequence must begin during the period of length *Δ*_*I*_ that starts when *X* is first observed interacting. This represents a situation in which *X* arrives in the system in an infectious state.

We have chosen to use reachability as it incorporates the relevant elements of disease dynamics while also being relatively fast to compute. It does, however, make implicit assumptions about the dynamics of the disease in question. Specifically, it is assumed that the disease variables, i.e. the latent and effective infectious period durations, are homogeneous across the population. To test whether the results obtained are sensitive to these assumptions, we perform disease simulations on the same data using a computation model.

### Measuring the effect of synchronization

Real diseases exhibit a range of dynamical behaviors that may invalidate the results obtained through the analytical approach of reachability. For example, there may be variation in the distribution of latent periods [38], the length of the effective infectious period (between onset of infectiousness and social withdrawal) [18], the proportion of individuals that are asymptomatic [39, 40], and the perseverance of individuals [13]. Here we describe how we use a computational disease model over the empirical contact network data to measure the impact of synchronization and its sensitivity to changes in each of these variables.

The disease simulation, based on an SEIR model (fully described in Section S1 and Figure S3 of the Additional file 1), takes as input: the temporal contact data, a “seed” individual from which the outbreak originates, the time for which the seed becomes infectious, and the following disease parameters: *β*, the per-second probability of transmission during contact between an infectious individual and a susceptible one; the mode of the latent period distribution, $\hat {\Delta }_{E}$; the dispersion of the distribution of latent periods $\sigma _{g}^{(E)}$; the mode of the effective infectious period distribution, $\hat {\Delta }_{I}$; perseverance of the population, 1/*k*_*I*_, which we define as the reciprocal of the shape parameter of the effective infectious period distribution and which captures the variation in host response to disease; and the proportion of exposed individuals that are asymptomatic, *a*, who do not experience symptoms or sickness behaviors, and thus experience a longer effective infectious period of *Δ*_*I*_=*Δ*_*J*_. By using the modes of the distributions of *Δ*_*E*_ and *Δ*_*I*_, and not the mean, we are able to vary the frequency of large outliers through dispersion and perseverance while keeping the outcome for the typical individual at a specific value.

According to our hypothesis, we expect the number of disease cases to be largest when the generation times of the outbreak are close to multiples of 24 h. The *generation time* for a transmission event is the time between the moment the host received the infection and the moment they transmitted it to another individual. Since the expected time between the onset of infectiousness and transmission is $\hat {\Delta }_{I}/2$ h (assuming infections times are distributed uniformly over the effective infectious period), the expected generation time is $\hat {\Delta }_{E}+\hat {\Delta }_{I}/2$. We therefore expect to find the largest outbreaks to occur in the simulation when $\hat {\Delta }_{E}=23$ and the smallest when $\hat {\Delta }_{E}=11$; we use the difference between these two cases to measure the effect of synchronization.

More specifically, we select each individual, *i*, in the population as the seed of the outbreak, and the time of their first contact as the start of their effective infectious period. The outbreak is allowed to proceed as per the SEIR model and is complete when there are no longer any infected individuals in the population, or when there are no more contacts in the population (due to the limitation of the datasets). This is repeated 100 times for each individual, *i*, with $\hat {\Delta }_{E}=11$ h and another 100 times with $\hat {\Delta }_{E}=23$ h. To measure the magnitude of the outbreak, we calculate the mean number of infected individuals across these simulations and denote this as $I\left (i,\beta,\hat {\Delta }_{E}, \hat {\Delta }_{I}, \Delta _{J}, \sigma _{g}^{(E)}, k_{I}, a\right)$. We note that we do not include the seed, or those infected directly by the seed, in this calculation so as to exclude infections that have no relation to the latent period. The *synchronization effect* for an individual *i* is defined as $\left [ I\left (i,\beta,\hat {\Delta }_{E}=23, \hat {\Delta }_{I}, \Delta _{J}, \sigma _{g}^{(E)}, k_{I}, a\right)- I\left (i,\beta,\hat {\Delta }_{E}=11, \hat {\Delta }_{I}, \Delta _{J}, \sigma _{g}^{(E)}, k_{I}, a\right) \right ]$.

#### Measuring disease impact with household contacts

Given the different structure of the urban contact network model, we specify further the disease simulation algorithm on this network. Here, the disease simulation begins with a seed becoming infectious at a random time during the first 24 h of the synthetic temporal contact network (which happens to be a Monday). To quantify the impact of household contacts on the synchronization effect, we consider three models: (I) No sickness behavior is implemented. Individuals are infectious for the entirety of the true infectious period, i.e. *Δ*_*I*_ = *Δ*_*J*_. We assume that the transmission probability, *β*, is lower in this case to make the results comparable to the remaining cases (see Additional file 1: Section S2.1). (II) Sickness behavior leads to social withdrawal from non-home contacts only. This withdrawal takes effect the day after infectiousness begins (details are described in Additional file 1: Section S2.1). Lastly, (III) extreme sickness behavior is implemented (as we did with the RFID data) and *Δ*_*I*_≪*Δ*_*J*_. Thus, infected individuals socially withdraw from all contacts (home and non-home).

## Results

In the three RFID contact networks, the rate of contact between individuals fluctuates periodically in time with the cycles of human social activity (see Fig. 2). By considering the spread of disease through these networks, we find that the impact of infectious disease is maximized when timing of infectiousness is synchronized with these temporal dynamics. We show this through analytical measurement of the temporal network structure (reachability) and test the robustness of these results through analysis of simulated disease outbreaks on each network. We further show that similar synchronization occurs for outbreaks on a larger scale, and with various types of contact, using the urban contact network data.

### Influence of the latent period on disease impact

To reveal the epidemiological impact of the timing of infectiousness, we computed the mean reachability (over all individuals in the network) for a range of latent periods and an effective infectious period of *Δ*_*I*_=2. The results are shown in Fig. 2.

For the conference setting we observe peaks in reachability when the latent period is 24 or 48 h. The peak corresponding to a latent period of 24 h is larger than the size of the peak corresponding to an approximate 48-h latent period. In the first case, those infected by the seed on day one, can cause a second generation of infections on day two, which then causes a third generation infections on day three. In the second case only two generations of infection are possible.

Similarly, in the hospital setting we observe four peaks in reachability. The largest peak corresponds to a 24-h latent period and represents generations of infection that reproduced on days 2-5 of the hospital contact dataset. The peak corresponding with a 48-h generation time represents a disease that was able to reproduce on only 2 of the 5 days (2 and 4 days after the time the seed was infectious). The final two peaks represent the optimal latent periods in cases where only 1 generation of infections is possible due to the limited time frame of the data.

Results for the school setting show a comparable pattern but on a larger time-scale. In this data, all individuals are seeded on day 1; the peak in reachability corresponding with a 24-h generation time includes infections that occurred on days 2-5; since no contacts occur on weekends, the disease dies out on Day 6 (Saturday). Peaks corresponding to generation times of 2 days and 4 correspond to outbreaks that can only survive for 2 generations of infection. In both of these scenarios, the disease also dies out over the weekend. The absolute maximum reachability corresponds to a latent period of 7 days. This peak occurs because infections caused by the seed on the first day are able to reproduce on the same day of the following week, a third generation can again occur on the same day of the next week, and this pattern can continue for all six weeks of the data. Epidemics with latent period durations that are not a multiple of 7 days will eventually die out because of the absence of social contact during the weekends.

### Robustness of the synchronization effect

To consider the sensitivity of the synchronization effect to realistic variability in pathogen or host behavior characteristics, we simulated disease outbreaks on the empirical contact data over a range of parameter values. For the initial baseline parameters, we assume a latent period dispersion factor of $\sigma _{g}^{(E)}=1.1$ which represents the low, yet realistic, value for respiratory infections [41]. We set the mode of the effective infectious duration distribution, $\hat {\Delta }_{I}=2$ h. (While we are unaware of empirical data measurements of *Δ*_*I*_, [42] report an average of 8.5 work hours are lost due to severe common colds). We set a true infectious period of *Δ*_*J*_=24 h, following [43]. We also assume that the asymptomatic proportion, *a*=0 and perseverance, 1/*k*_*I*_=0.2.

**Fig. 3:**
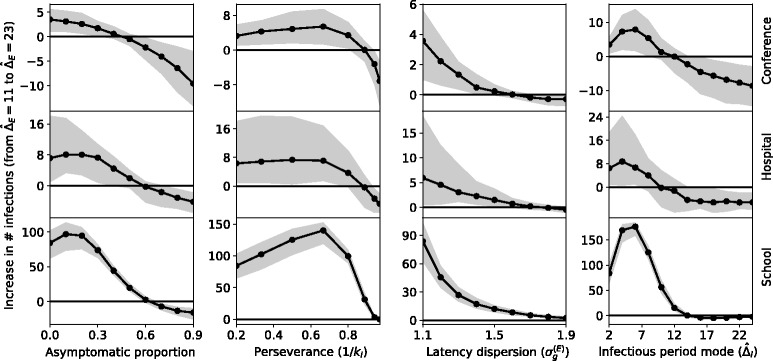
The effect of synchronization. The dark line represents the median effect size over individuals in the population. The effect size is defined as the increase in mean outbreak size between a disease for which the latent periods follow a log-Normal distribution with a mode of 11 h, and one which has a mode of 23 h (dispersion factors are equal). Points correspond to the values on the horizontal axis for which the effect size was computed and the gray area is the inter-quartile range. We see that the synchronization effect observed in “Influence of the latent period on disease impact” Section is present for a wide range of parameters values in the disease model

In this baseline case, the synchronization effect was positive, i.e. changing the mode value of the latent duration distribution from $\hat {\Delta }_{E}=11$ to $\hat {\Delta }_{E}=23$ yielded an increase in the number of infections. This is consistent with the results of the previous section (Influence of the latent period on disease impact). We then tested the robustness of this effect by perturbing each parameter away from its baseline value towards more realistic scenarios (Fig. 3). The following paragraphs report the effect of each parameter perturbation.

Since asymptomatic individuals remain in the system for significantly longer than those who do experience symptoms, their presence increases the overall outbreak size. Moreover, since the time at which they first become infectious has relatively little affect on the number of infections they cause, we expect to see a decrease in the synchronization effect as *a* increases. This was found to be the case for moderate proportions of asymptomatic individuals (0.1<*a*<0.3); however, at lower values, the presence of asymptomatic individuals did not benefit the non-synchronized disease significantly more than the synchronized one. This is seen most clearly in the school setting, in which the synchronization effect was actually amplified by the addition of asymptomatic cases. The asymptomatic proportion for influenza has been estimated to be within 4−28*%* [44], making the relevance of synchronization for influenza unclear.

Increasing perseverance (i.e. the variation in the time to social withdrawal among infectious individuals) leads to longer periods of infectiousness for some individuals, and consequently allows for more opportunities for transmission. In Fig. 3, we see that the relationship between perseverance and synchronization effect is qualitatively similar to that seen for the asymptotic proportion; at low values, perseverance benefits both the non-synchronized and synchronized diseases. For larger values, the individuals who persevere for prolonged durations dominate transmission and the time at which they first become infectious loses significance.

Dispersion in the distribution of latent periods, $\sigma _{g}^{(E)}$, affects the mean outbreak size differently depending on the value of $\hat {\Delta }_{E}$. For $\hat {\Delta }_{E}=11$, as dispersion increases, the probability that the latent period will be close to 11 gets smaller and the probability that the effective infectious period will intersect with a period of high social activity gets larger. The opposite is true for $\hat {\Delta }_{E}=23$. Increasing $\sigma _{g}^{(E)}$ therefore causes the difference in mean outbreak sizes of the two cases to converge towards zero.

As the effective infectious period, $\hat {\Delta }_{I}$, increases, there are two consequences: outbreaks become larger as there is more time for transmission, and the exact duration of the latent period, $\hat {\Delta }_{E}$, becomes less significant since infected hosts spend a larger fraction of a 24-h period in an infectious state (and are thus more likely to have social interactions while infectious, regardless of when their infectiousness began). Consequently, we find that the synchronization effect is maximized at approximately $\hat {\Delta }_{I}=6$ h; here, the effective infectious period is long enough for a relatively large number of infections to occur, but short enough that it can be entirely absorbed by periods of low activity in the asynchronous case.

In the conference and hospital settings, the number of generations of infection is limited by the duration of the data (3 and 4 days, respectively). In general, this gives the shorter latent period an advantage over the longer one, and we eventually see the synchronization effect go below zero. In the primary school dataset, on the other hand, most infections die out before the end of the 6-week duration and the synchronization effect is rarely negative.

### Effect of household contacts

Since the hospital, school, and conference datasets do not include interactions that occur outside of their established settings, it is necessary to ask whether the synchronization effect would be found in a complete contact network which included long-duration home contacts during evenings and weekends. The effect of these missing links could be significant enough to break the synchronization pattern we see. For example, interactions that occur in the household may act as bridges that allow infection to spread between schools and workplaces [45], contributing more to the size of the outbreak than does the effect of synchronization. We present the results of our epidemic simulations on the urban contact network model to address this question.

**Fig. 4:**
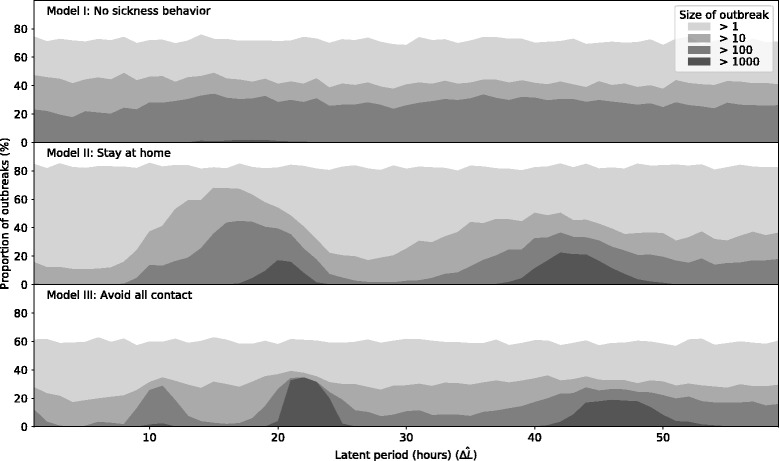
Simulated outbreaks in a synthetic urban environment The proportion of outbreaks that exceed a given size are shown (for each latent period 10^3^ simulations were performed starting from randomly selected seeds at random times during the first day). The three models shown are described in “Effect of household contacts” Section

In the first model (I), we consider epidemic outcomes when infected individuals do not change their behavior in any way and *Δ*_*I*_=*Δ*_*J*_=24 h. In Fig. 4, we see that the duration of the latent period has little effect on the disease outcomes. However, even in the absence of sickness behavior, epidemics are more likely to occur at values around 18 h.

In the second model (II) illness does not affect household contacts. We assume that if an individual becomes symptomatic during home hours, they will stay at home; and if the individual becomes symptomatic during the daytime, they will continue to engage in non-household social contacts until the end of the day. Epidemics occur when the latent period is close to 20 and 42 h. We note that large epidemics are likely across a larger range of latent period modes in the second cluster due to the larger variance in the distribution of latent periods. This is a consequence of the fact that incubation (and thus latent) periods follow a log-Normal distribution for which higher mean results in a higher variance [46].

The third model (III) is similar to that used in the reachability analysis, where infected individuals withdraw from all contacts after 2 h of infectiousness. This results in a similar synchronization pattern as model II, however, as we might expect from a typically shorter infectious period, the latent period durations that correspond to epidemics are more tightly clustered (around 23 and 46 h).

## Discussion

The reproductive potential of a pathogen is driven by the host social behavior that underlies transmission. We have considered the class of diseases that causes hosts to socially withdraw due to symptoms, and focused on the role that the latent period plays in determining their epidemic potential. Our results support the hypothesis that disease risk can be amplified by synchronization between the latent period of the infection and the circadian rhythms of the host population.

Through analysis of empirical face-to-face contact networks, we have shown that a disease is most pervasive when its generation time is close to a multiple of 24 h. In such cases, infections that occur during a socially busy time of day will likely cause another generation of infections during the same socially active time on a subsequent day, thus perpetuating the life-cycle of the pathogen. Conversely, we observe minimum disease risk when the generation time is out-of-phase with human circadian rhythms by exactly half a day. In this case, individuals infected during socially busy times will become infectious during a time when there are fewer opportunities for transmission.

The results of our final analysis illustrate that the avoidance or non-avoidance of contact with household members in the evening has limited effect on the optimal generation time of the disease. In all models of that analysis, a significant fraction of transmission occurs through household contact (53%, 67% and 44% for models I, II and III, respectively) suggesting that the the period of social inactivity during sleeping hours is the main opponent to the disease (and the reason why epidemics die out faster when the latent period is not synchronized). This is most apparent in Models II and III, which could be said to be more virulent than Model I (higher transmission probabilities and more likely to provoke social withdrawal). In the trade-off between transmissibility and life history traits, this result suggests a novel advantage to being less virulent [47].

Our modeling approach has a number of limitations. Biologically, we have assumed that the population is homogeneously and perfectly susceptible. While this assumption would be invalidated with pre-existing natural or vaccine-induced immunity in the host population, we expect our results to hold given any periodicity in the contact patterns of the remaining susceptible population. Additionally, while our results indicate that synchronization with human circadian cycles should be advantageous for an infectious disease, this is only true when the window of opportunity for transmission is suitably short. In some cases symptoms coincide with infectiousness closely enough to induce a rapid behavioral response from the host and be consistent with our results [11, 12, 48], while others exhibit much longer periods of pre-symptomatic viral shedding that would be unaffected by human circadian rhythms [23]. However, even in these cases there is doubt that transmission occurs before the onset of symptoms [49]. Another possibility that would invalidate our model is if infectiousness persists after the end of symptoms but evidence suggests that this does not occur for influenza [11].

Behaviorally, we make a number of simplifying assumptions about social distancing and contact patterns. For one, we assume that behavior change by infected individuals eliminates transmission potential entirely, but there may be more variation in this change. Second, we ignore that social isolation could also result from susceptible individuals avoiding symptomatic infected individuals; these responses may be driven by public awareness of the epidemic and therefore change dynamically as it grows in size [50]. Third, the contact network data we use in this study do not represent the range of human social behavior heterogeneity: the RFID contact networks neglect any influence from outside the selected location, and the urban contact network model assumes temporal homogeneity in contact dynamics. In combination, however, our results do provide qualitative insights into the impact of partial social withdrawal.

The results of this analysis suggest that by having a life-cycle that synchronizes with the circadian cycles of human behavior a pathogen can gain a reproductive advantage over those that do not. If this hypothesis holds, we would expect to see the generation times of diseases to be close to multiples of 24 h. A recent review of serial intervals (which are closely related to generation times if symptoms and infectiousness track each other) reported the mean and standard deviation from seven influenza studies [51]. In all reported cases, the mean generation time was within 0.3 days of perfect synchronization; the probability that this would occur by chance is less than 0.1. Moreover, five of these cases were out of phase by 0.2 days or less, and the two that were further from synchronization had variances that were above the average. These data are consistent with the idea that for influenza to reach epidemic scale, its generation time must either synchronize with human circadian rhythms, or have highly variable latent period durations.

The conclusions of this work have implications for predicting and controlling infectious disease transmission at the population scale. Contact rates vary dynamically, both periodically according to cycles of human behavior, and in response to the disease itself. Consequently, the window of opportunity for transmission may be much shorter than the actual duration of infectiousness determined in experimental studies; thus, the important question is not *How long is the infectious period?*, but rather *When does the infectious period begin?*.

Epidemic models that do not consider the effect of sickness behaviors, and assume a constant (non-cyclic) rate of contact, may lead to misleading conclusions about the drivers of transmission. This is most likely to apply to cases where sickness behaviors are strong and infectiousness begins shortly before the onset of symptoms. Previous work has drawn attention to the importance of this period, suggesting that control strategies involving the treatment or isolation of symptomatic individuals are only effective if individuals can acknowledge that they have the disease within a reasonable amount of time [18]. Our work suggests that, in such cases, the duration of the latent period becomes a primary driver of transmission patterns.

Finally, we suggest the possibility that control strategies for managing infectious disease may be designed around the effects of synchronization. For example, antivirals for influenza are expected to alleviate symptoms if taken within 24 h of symptom onset. Our results suggest that if antivirals eliminate transmissibility (which does not appear to be supported for influenza, [52]), it would be possible to optimally time antiviral administration to minimize cases by making the period from exposure to antiviral onset out of phase with human circadian rhythms. As another example, the manipulation of school and workplace schedules to break the synchronization effect may result in a hostile environment towards infections with particular latent periods. More generally, a better understanding of the coupling between human and disease dynamics could lead to methods of social distancing that are sensitive to the temporal dynamics of infectious disease.

## Conclusions

We have proposed the hypothesis that infectious diseases are most pervasive when their latent period, and consequently their generation times, are synchronized with circadian rhythms of the host population. We predict that synchronization is most likely to occur for diseases that invoke a strong symptomatic response and lead to a withdrawal of social contact in the host. Our analysis of empirical temporal contact networks supports this argument by showing that the reachability of a disease is strongly dependent on its latent period and that simulated diseases that synchronize with the daily rhythms of social contact gain a reproductive advantage over those that do not. We conclude that the surveillance, modeling, and mitigation of infectious diseases should carefully consider the consequences of disease synchronization, and, more broadly, the complex interactions that can occur between social and biological systems.

## Additional file

Additional file 1 Supplementary information. (PDF 904 kb)
