# Supplementary material for: The reachability of contagion in temporal contact networks: how disease latency can exploit the rhythm of human behavior
Source: BMC Infect Dis. 2018 May 15;18:219. doi: 10.1186/s12879-018-3117-6 (PMC5952858; doi:10.1186/s12879-018-3117-6)
Supplement: Supplementary file 1 — Supplementary information. (PDF 904 kb) [file 12879_2018_3117_MOESM1_ESM.pdf]

Supplementary information

S1 Simulating the spread of disease on temporal network data

The backbone of the SEIR simulation algorithm is shown in Figure S3. Here we give details about the parameters in the model and describe additional features that are not shown in the figure.

S1.1 Transmissibility β

For the RFID data, the value of β is calculated for each data set separately in a way that makes the outcome of the simulation sensitive to the other parameters and not dominated by a β value that is too large (causing every simulation to be an epidemic) or small (causing all outbreaks to die-out in one generation). This is achieved by choosing β such that a typical individual infected at a random point in time in a otherwise fully susceptible population is expected to infect exactly 1 other. This can be calculated by solving

$$\frac{\text{Total interaction duration}}{\text{Population size} \times \text{Total duration}} \times \hat{\Delta}_I \times \beta = 1$$

The quotient on the left hand side is the probability that the individual will be engaged in contact. This is then multiplied by the number of seconds for which they remain infectious (in all cases we use $\hat{\Delta}_I = 2$), and again by the probability that transmission will occur during any one of those seconds. This gives $\beta = 0.0049$ for the conference data, $\beta = 0.0019$ for the hospital, and $\beta = 0.0009$ for the primary school.

Note that this method involves averaging over the entire duration of the data, including periods of both high and low activity. In practice, if the host is infectious during a high activity period then the the number of secondary infections we would expect them to cause will be significantly higher than 1. Conversely, if are infectious during a time of low activity then it will be significantly lower.

S1.2 Asymptomatic proportion a

Some members of the population may show no signs of infection (up to 28% reported for influenza [1] and 32% for rhinovirus [2]), or might just ignore them completely, in which case their behavior does not change. At the beginning of the simulation, a random sample of the population are chosen to be asymptomatic. These individuals, who make up a fraction a of the total population, have an infectious period of 24 hours. We also acknowledge that immunocompromised individuals are asymptomatic and infectious for extremely long periods of time [3], however, we consider these cases to be too rare to incorporate into the model.

S1.3 Latent period dispersion $\sigma_g^{(E)}$

The duration of the latent period may vary between individuals depending on their age, gender, or other characteristics [4–7]. While the latent period and the incubation period are not the same, we assume that the biological factors determining their length to be similar, i.e. the processes described in [8], and thus we assume that the distribution of latent periods is log-Normal [9]. In the simulation, the latent duration for each infected individual is drawn from a log-Normal distribution with mode $\hat{\Delta}_E$ and dispersion factor $\sigma_g^{(E)}$ (the geometric standard deviation of the distribution). We use $\sigma = \sigma_g^{(E)}$ and $\mu = \sigma^2 + \log(\hat{\Delta}_E)$ to get the standard parameters for the log-Normal distribution.

S1.4 Perseverance $1/k_I$

Once infected, the behavioral response of individuals may vary; some might leave the system (or take other measures to prevent infection) immediately, whereas some may remain a risk to others for a more prolonged duration [10]. In the simulation, the duration of the infectious period of each individual is randomly selected from a gamma distribution with a mode of $\hat{\Delta}_I$ hours. We define perseverance as $1/k_I$ where k_I is the shape parameter of the gamma distribution. By choosing the scale parameter of the Gamma distribution to be $\theta = \hat{\Delta}_I/(k_I - 1)$ we ensure that the mode does not change while increasing the perseverance fattens the the tail of the distribution.

S2 Features for urban population model

S2.1 Transmissibility β^{h} and β^{w}

For the urban contact network model, beta was chosen so that the run-time of the three sickness behavior scenarios were approximately equal. This corresponds to an approximately equal number of infections occurring in all three scenarios across all latent period durations.

Additionally, since fewer contacts ocurred during the evening (mean degree at home $\bar{k}^{\mathrm{h}} = 2.28$) than there are during the morning and afternoon hours ($\bar{k}^{\mathrm{w}} = 14.15$ away from home), we expect the intensity (or frequency) of these interactions to be higher. To achieve this, each information about the location of each contact was used; we set the transmission rate to be higher for household contacts than for contacts that occur elsewhere. In particular, we set the two different values of the transmission probabilities, β^{h} and β^{w}, so that $\bar{k}^{\mathrm{h}}\beta^{\mathrm{h}} = \bar{k}^{\mathrm{w}}\beta^{\mathrm{w}} = \beta$.

For the analysis presented we chose $\beta = 2.3 \times 10^{-5}$, $\beta = 1.3 \times 10^{-4}$, $\beta = 3.6 \times 10^{-4}$ for models I, II and III, respectively.

S2.2 Sickness behavior

To incorporate the idea that sickness behavior might not depend on a fixed amount of time but instead depend on how one feels when they wake up in the morning, we added the following dynamic to the simulation: if the onset of infectiousness happens between 8am and 4pm on any given day, then the individual will remain at work until 4pm but beyond this time all work contacts will be ignored by the simulation. If infectiousness begins at a time outside of this range, all of their work contacts are ignored by the simulation.

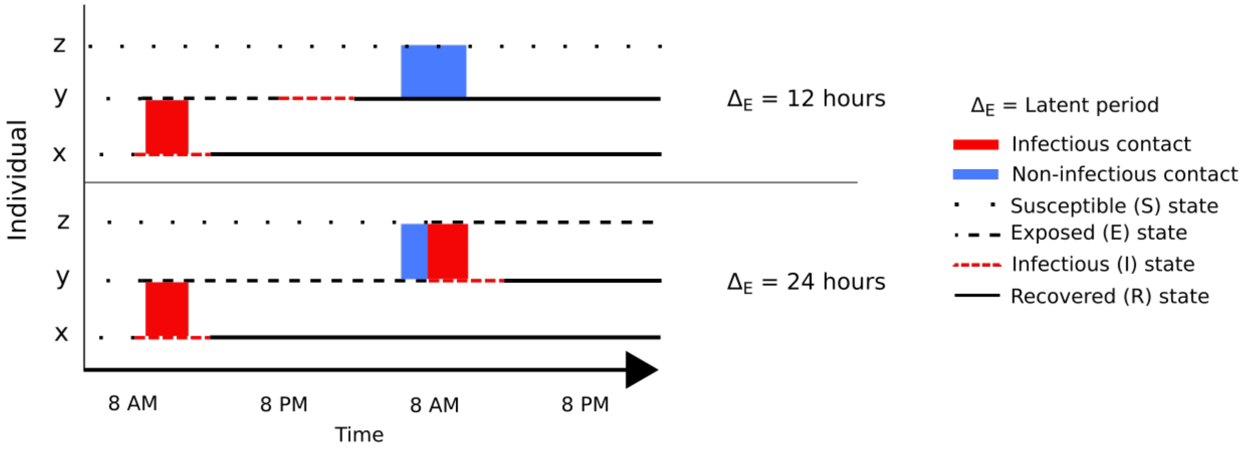

Figure S1: An example showing the difference between a 12 and 24 hour latent period. For both scenarios, we consider the same contact sequence. The color and pattern of the line represent the disease state of the individual. The color of the shaded region between two lines represents an either a potentially interaction (in the bottom panel the infectious period of Y begins during the interaction between Y and Z). Individual X is infected on the morning of the first day. When infected with a disease with a 24-hour latent period, the infection can reach both individuals Y and Z; however, with a 12-hour latent period, it can only reach individual Y.

References

[1] N. H. Leung, C. Xu, D. K. Ip, and B. J. Cowling, "Review article: The fraction of influenza virus infections that are asymptomatic: A systematic review and meta-analysis.," 2015.

[2] S. E. Jacobs, D. M. Lamson, K. S. George, and T. J. Walsh, "Human rhinoviruses," *Clinical microbiology reviews*, vol. 26, no. 1, pp. 135–162, 2013.

[3] N. Lehners, J. Tabatabai, C. Prifert, M. Wedde, J. Puthenparambil, B. Weissbrich, B. Biere, B. Schweiger, G. Egerer, and P. Schnitzler, "Long-term shedding of influenza virus, parainfluenza virus, respiratory syncytial virus and nosocomial epidemiology in patients with hematological disorders," *PLOS ONE*, vol. 11, pp. 1–17, 02 2016.

[4] S. Blythe and R. Anderson, "Distributed incubation and infectious periods in models of the transmission dynamics of the human immunodeficiency virus (hiv)," *Mathematical Medicine and Biology*, vol. 5, no. 1, pp. 1–19, 1988.

[5] A. L. Lloyd, "Destabilization of epidemic models with the inclusion of realistic distributions of infectious periods," *Proceedings of the Royal Society of London B: Biological Sciences*, vol. 268, no. 1470, pp. 985–993, 2001.

[6] A. L. Lloyd, "Realistic distributions of infectious periods in epidemic models: changing patterns of persistence and dynamics," *Theoretical population biology*, vol. 60, no. 1, pp. 59–71, 2001.

[7] H. J. Wearing, P. Rohani, and M. J. Keeling, "Appropriate models for the management of infectious diseases," *PLOS Medicine*, vol. 2, 07 2005.

[8] B. Ottino-Loffler, J. G. Scott, and S. H. Strogatz, "Evolutionary dynamics of incubation periods," *eLife*, vol. 6, p. e30212, dec 2017.

[9] P. E. Sartwell *et al.*, "The distribution of incubation periods of infectious disease.," *American Journal of Hygiene*, vol. 51, pp. 310–318, 1950.

[10] K. Van Kerckhove, N. Hens, W. J. Edmunds, and K. T. D. Eames, "The impact of illness on social networks: Implications for transmission and control of influenza," *American Journal of Epidemiology*, vol. 178, no. 11, p. 1655, 2013.

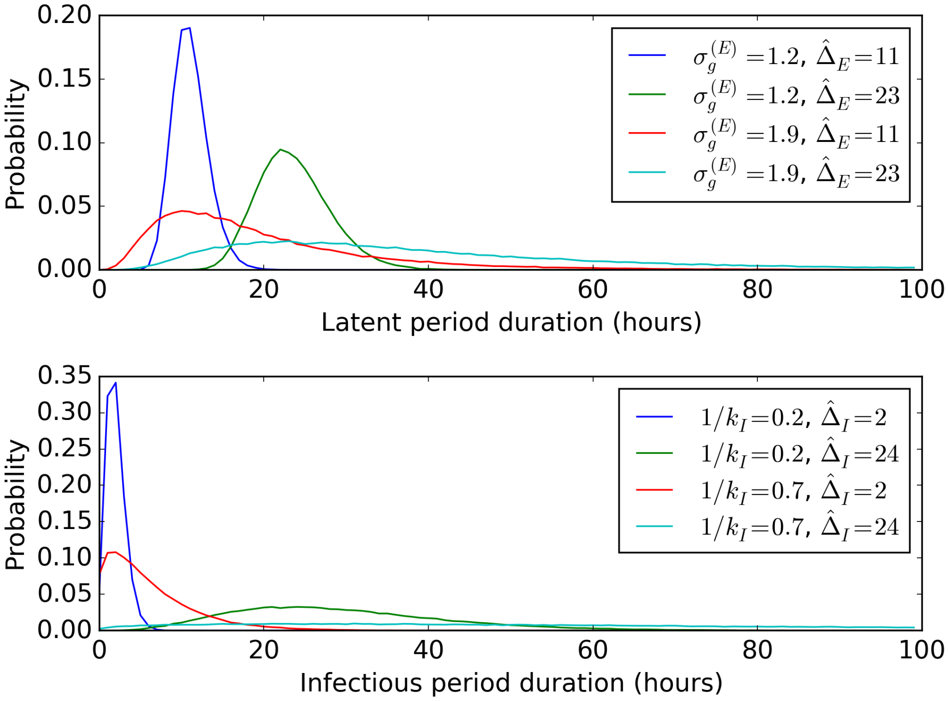

Figure S2: Probability distributions for different parameter combinations. In the disease simulation, latent periods follow a log-Normal distribution and infectious periods follow a gamma distribution. To illustrate various parameter combinations to provide a visual guide to understanding the meaning of the paramters in the model.

[11] J. M. Harris and J. M. Gwaltney, "Incubation periods of experimental rhinovirus infection and illness," *Clinical infectious diseases*, vol. 23, no. 6, pp. 1287–1290, 1996.

[12] J. Lessler, N. G. Reich, R. Brookmeyer, T. M. Perl, K. E. Nelson, and D. A. Cummings, "Incubation periods of acute respiratory viral infections: a systematic review," *The Lancet infectious diseases*, vol. 9, no. 5, pp. 291–300, 2009.

[13] F. Carrat, E. Vergu, N. M. Ferguson, M. Lemaitre, S. Cauchemez, S. Leach, and A.-J. Valleron, "Time lines of infection and disease in human influenza: a review of volunteer challenge studies," 2008.

[14] L. Canini and F. Carrat, "Population modeling of influenza a/h1n1 virus kinetics and symptom dynamics," *Journal of virology*, vol. 85, no. 6, pp. 2764–2770, 2011.

[15] A. Cori, A. Valleron, F. Carrat, G. S. Tomba, G. Thomas, and P. Boëlle, "Estimating influenza latency and infectious period durations using viral excretion data," *Epidemics*, vol. 4, no. 3, pp. 132–138, 2012.

[16] "Webmd medical reference."

[17] "Mayo clinic."

[18] "Centers for disease control and prevention."

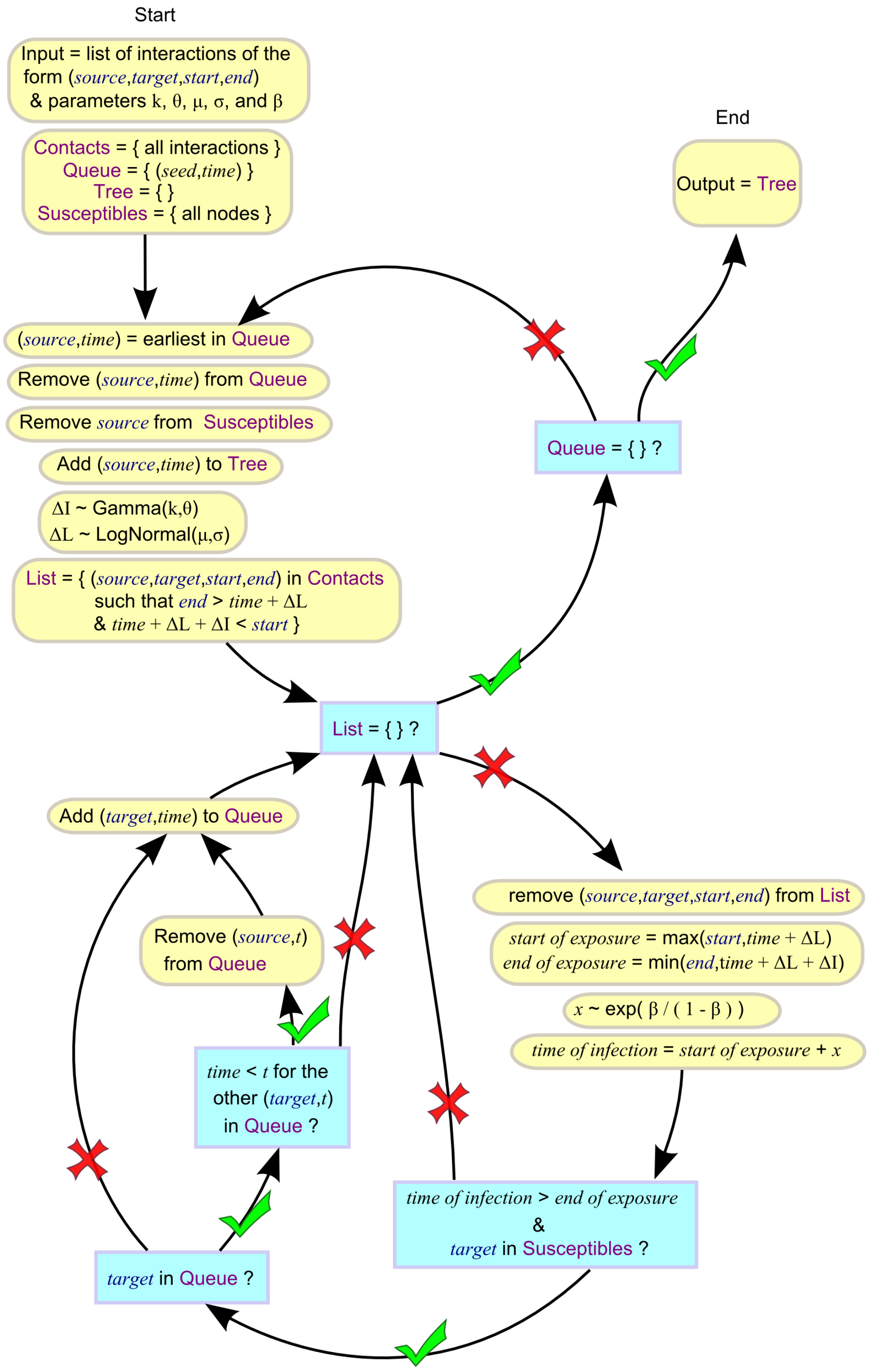

Figure S3: The backbone simulation SEIR model for temporal network data. Details of the meaning of the parameter values are found in Section S1. Arrows show the order in which operations are to be completed. Blue boxes represent conditional rules of the algorithm; green ticks indicate the next operation if the answer is yes, red crosses if the answer is no.

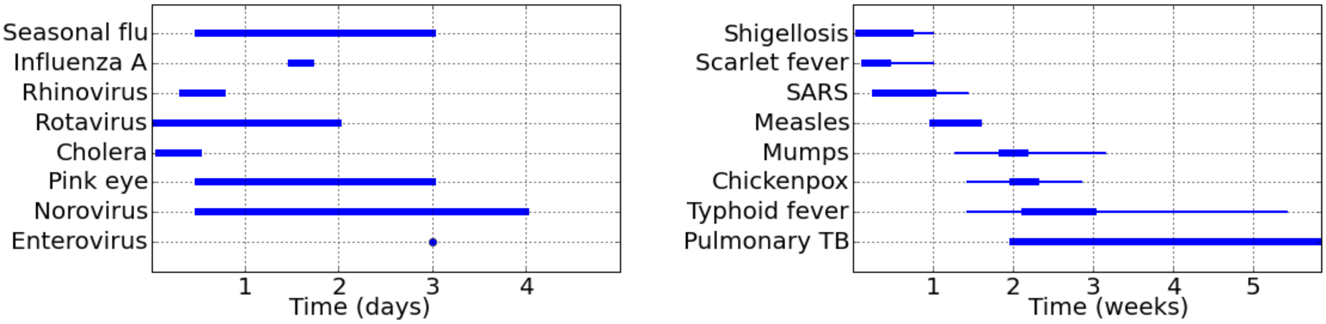

Figure S4: Latent periods of infectious diseases based on information gathered from the literature. The bold line represents range of possible latent periods that can be estimated with high certainty. The thin line represents that the latent period could be longer or shorter but there is disagreement among sources. Time is in days for the left panel and in weeks for the right. The latent period range for pulmonary TB was shortened for ease of viewing (actual upper bound is 12 weeks). All information shown, as well as incubation and infectious periods, are provided in Table S1.

Table S1: The maximum range of latent, incubation, and infectious periods, where available, for several transmissible diseases, as collected from the literature.

**Disease**	**Latent period**	**Infectious period**	**Incubation period**
Rhinovirus	8 - 18 hours [11]	7 - 14 days [2]	1.4 - 2.4 days [12] or 16 hours [11]
Seasonal flu [13]	0.5 - 3 days, average 1.1 days	4.31 - 5.29 days, average 4.8 days	1 - 3 days
Influenza A (H1N1) [14]	0.7 - 1.9 days mode 2.2 days	1.7 - 7.0 days, average 4.8 days	1.0 - 2.4 days
Influenza A (H1N1 and H3N2) [15]	1.5 - 1.7 days, mean 1.6 days	0.5 - 1.7 days, mean 1.0 day	not provided
Pink eye (bacterial) [16, 17]	24-72 hrs	7 - 10 days or until 24 hours after start of antibiotics	24-72 hrs
Pink eye (viral) [16,17]	12 - 72 hour	usually 5 - 7 days, can become chronic	12 - 72 hour
Enterovirus D68 [18]	3 days	1 - 3 weeks, average 10 days	3 - 6 days, others say 3 - 10 days
Rotavirus [16,18]	48 hours or less	6 - 11 days	48 hours
Scarlet fever [16]	12 hours - 7 days	1 - 2 weeks	12 hours - 7 days, usually 2 - 5 days
Cholera [16,18]	2 hours	7 - 14 days	2 hours 5 days, usually 2 - 3 days
Norovirus [18]	12 - 94 hours	4 - 6 days	12 - 48 hours
Shigellosis [18]	usually 1 - 3 days, up to 7 days	2 days up to “a few weeks”	usually 1 - 3 days, up to 7 days
SARS [18]	usually 2 - 7 days, up to 10 days	2 - 21 days	usually 2 - 7 days, up to 10 days
Measles [18]	6 - 15 days	9 days	7 - 14 days (cold-like symptoms), 10 - 19 days (rash)
Chickenpox [16]	10 - 21 days	6 - 8 days	10 - 21 days
Mumps [18]	9 - 22 days, usually 13 - 15 days	8 days	12 - 25 days, usually 16 - 18 days
Typhoid fever [18]	10 days to about 1 week after symptoms onset	usually 2 weeks, can be up to 1 year	usually 15 - 21 days
P. tuberculosis [17,18]	2 - 12 weeks	2 weeks	2 - 12 weeks
